# The stem cell factor/c-*kit *receptor pathway enhances proliferation and invasion of pancreatic cancer cells

**DOI:** 10.1186/1476-4598-5-46

**Published:** 2006-10-18

**Authors:** Akira Yasuda, Hirozumi Sawai, Hiroki Takahashi, Nobuo Ochi, Yoichi Matsuo, Hitoshi Funahashi, Mikinori Sato, Yuji Okada, Hiromitsu Takeyama, Tadao Manabe

**Affiliations:** 1 Department of Gastroenterological Surgery, Nagoya City University Graduate School of Medical Sciences, Nagoya, 4678601, Japan

## Abstract

**Background:**

The transmembrane protein c-*kit *is a receptor tyrosine kinase (KIT) and KIT is expressed in solid tumors and hematological malignancies such as gastrointestinal stromal tumor (GIST), small-cell lung cancer and chronic myelogenous leukemia (CML). KIT plays a critical role in cell proliferation and differentiation and represents a logical therapeutic target in GIST and CML. In pancreatic cancer, c-*kit *expression has been observed by immunohistochemical techniques. In this study, we examined the influence of c-*kit *expression on proliferation and invasion using five pancreatic cancer cell lines. In addition, the inhibitory effect of imatinib mesylate on stem cell factor (SCF)-induced proliferation and invasion was evaluated. Finally, we also analyzed KIT and SCF expression in pancreatic cancer tissues using immunohistochemistry and correlated the results with clinical features.

**Results:**

RT-PCR revealed that two pancreatic cancer cell lines, PANC-1 and SW1990, expressed c-*kit *mRNA. By Western blot analysis, c-*kit *protein was also present in those lines. In KIT-positive pancreatic cancer cell lines, proliferation and invasion were significantly enhanced by addition of SCF. In contrast, SCF did not enhance proliferation and invasion in the three KIT-negative lines (BxPC-3, Capan-2 and MIA PaCa-2). 5 μM imatinib mesylate significantly inhibited SCF-enhanced proliferation to the same extent compared with the control. Similarly, SCF-enhanced invasive ability was significantly inhibited by 5 μM imatinib mesylate. KIT was expressed in 16 of 42 clinical specimens by immunohistochemistry, and KIT expression was significantly related to venous system invasion. Furthermore, patients expressing both KIT and SCF had a somewhat lower survival.

**Conclusion:**

Our results demonstrated that the SCF-KIT pathway enhanced the proliferation and invasiveness in KIT-positive pancreatic cancer cell lines and that the enhanced proliferation and invasion were inhibited by imatinib mesylate. We propose that inhibitors of c-*kit *tyrosine kinase receptor have the potential to slow the progression of KIT-positive pancreatic cancers.

## Background

The transmembrane protein c-*kit *is a receptor tyrosine kinase (KIT) which is closely related to other receptors including platelet-derived growth factor receptor and macrophage growth factor receptor [1,2]. The primary ligand for KIT is stem cell factor (SCF) which is also known as mast cell growth factor, steel factor and kit ligand [3-6]. Binding of SCF to KIT causes dimerization, autophosphorylation and signal transduction [7]. The SCF-KIT signaling system supports the proliferation, differentiation and survival of KIT-expressing cells, such as hematopoietic progenitors, mast cells, melanocytes, germ cells and cells of Cajal [8-12]. KIT is expressed in normal cells and also in solid tumors and hematological malignancies such as gastrointestinal stromal tumor (GIST) [13], small-cell lung cancer [14], colorectal cancer [15], Ewing's tumor [16], chronic myelogenous leukemia (CML) [17], neuroblastoma [18] and mast cell leukemia [19]. On the other hand, breast cancer and thyroid cancer are associated with loss of KIT expression [20,21]. Mutations are observed in c-*kit *in some malignant diseases such as GIST [13], chronic myelogenous leukemia [17] and mast cell leukemia [22]. Mutation of c-*kit *can result in activation of the receptor in the absence of ligand. Certain *c-kit *mutations are associated with more frequent relapse and decreased survival [23]. Thus, it is apparent that KIT plays a critical role in cell proliferation and differentiation [24,25] and represents a logical therapeutic target in GIST, CML and other diseases [26]. For example, c-*kit *tyrosine kinase receptor is targeted by imatinib mesylate (STI571, Glivec) [27].

In pancreatic cancer, c-*kit *expression has been observed by immunohistochemical techniques [28-30]. Furthermore, SCF may play a role in growth regulation in the normal pancreas [31]. However, at this time, the contribution of c-*kit *receptor to *in vitro *models of pancreatic cancer is not known. In this study, we examined the influence of c-*kit *expression on proliferation and invasion using five pancreatic cancer cell lines. In addition, the inhibitory effect of imatinib mesylate on SCF-induced proliferation and invasion was evaluated. Finally, we also analyzed KIT and SCF expression in pancreatic cancer tissues using immunohistochemistry and correlated the results with clinical features.

## Results

### Expression of c-*kit *in pancreatic cancer cell lines

**Figure 1:**
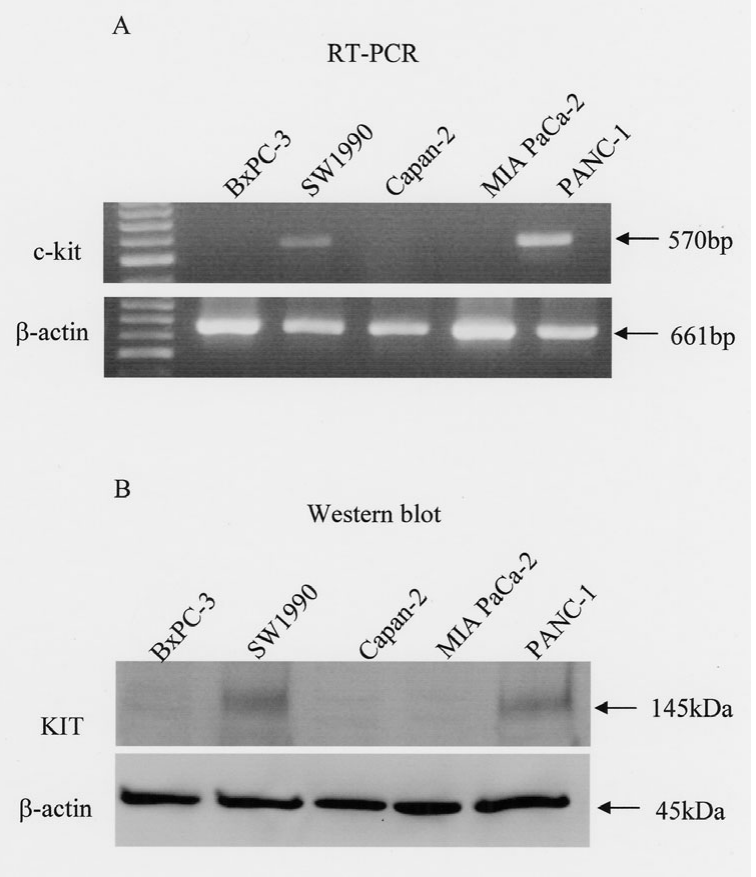
**c-*kit *mRNA and protein expression in pancreatic cancer cell lines**. (A) c-kit mRNA expression in pancreatic cancer cell lines was detected by RT-PCR. As a control, β-actin expression was also examined. (B) c-*kit *protein expression in pancreatic cancer cell lines was confirmed by Western blot analysis. Reprobing with an anti-β-actin antibody served as a control.

RT-PCR revealed that two pancreatic cancer cell lines, PANC-1 and SW1990, expressed c-*kit *mRNA (Fig. 1A). By Western blot analysis, c-*kit *protein was also present in those lines (Fig. 1B). Neither c-*kit *mRNA nor protein was detected in the other three pancreatic cancer cell lines, BxPC-3, Capan-2 and MIA PaCa-2. Thus, Western blot analysis confirms the PT-PCR results.

### Effect of SCF on pancreatic cancer cell proliferation

**Figure 2:**
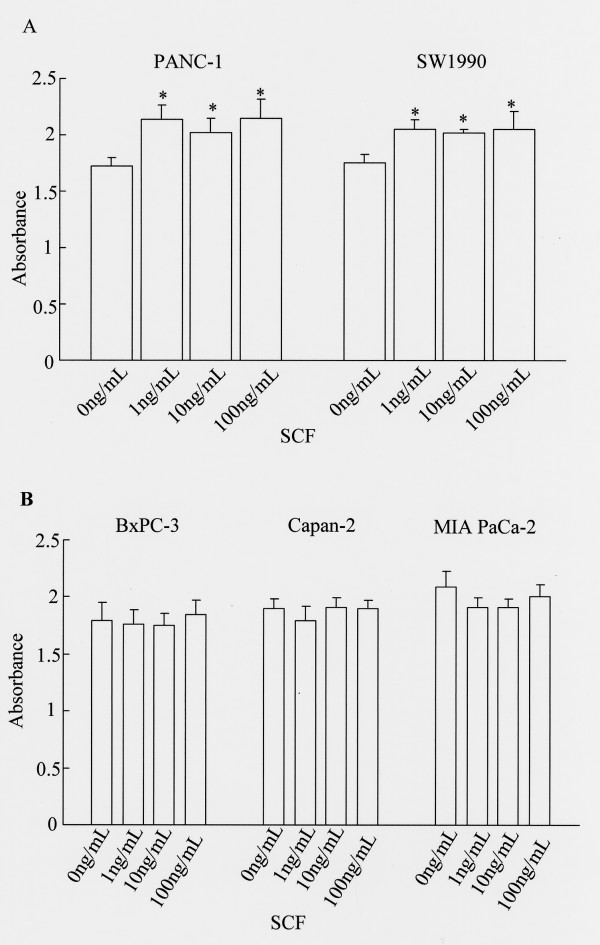
**Effect of SCF on proliferation of pancreatic cancer cell lines**. (A) KIT-positive pancreatic cancer cell lines, and (B) KIT-negative cell lines. Cell proliferation was determined using the WST-1 assay. SCF stimulated the proliferation of KIT-positive pancreatic cancer cell lines. Statistical analysis was performed by one-way ANOVA with the Dunnett test. Bars indicate the SD, *, p < 0.01 compared with control.

Figure 2 shows the effect of SCF on proliferation of KIT-positive and KIT-negative pancreatic cancer cell lines. In the two KIT-positive cell lines (PANC-1 and SW1990), proliferation was significantly enhanced by KIT ligand SCF when the concentration exceeded 1 ng/mL (Fig. 2A). In contrast, SCF did not enhance proliferation in the three KIT-negative lines (BxPC-3, Capan-2 and MIA PaCa-2) (Fig. 2B).

### Effect of SCF on pancreatic cancer cell invasion

**Figure 3:**
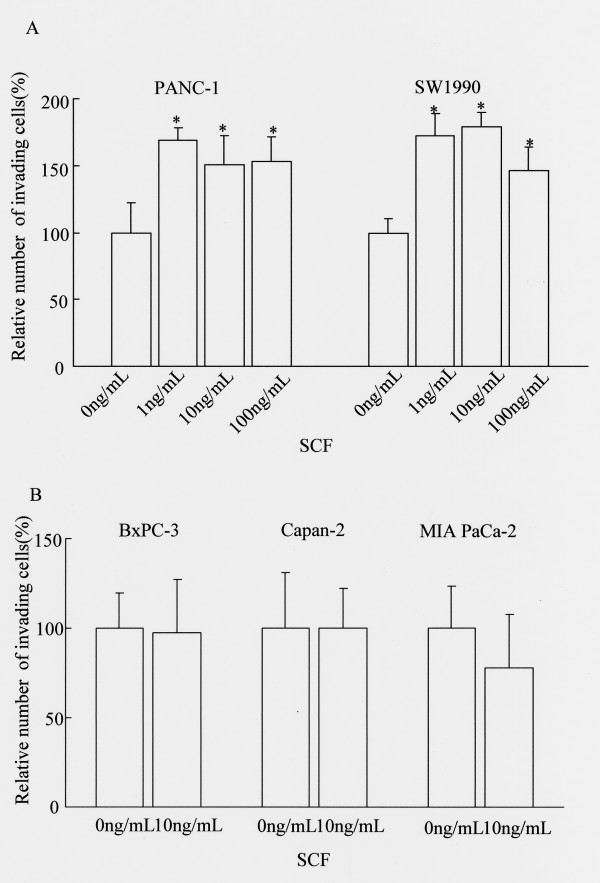
**Effect of SCF on invasiveness of pancreatic cancer cell lines**. (A) KIT-positive pancreatic cancer cell lines and (B) KIT-negative cell lines. Cell invasion was evaluated using the Matrigel assay. SCF enhanced the invasion in c-kit-positive pancreatic cancer cell lines. Statistical analysis was performed by one-way ANOVA with the Dunnett test for PANC-1 and SW1990 and by the Student *t *test for BxPC-3, Capan-2 and MIA PaCa-2. Bars indicate the SD, *, p < 0.01 compared with control.

Figures 3A and 3B show the effect of SCF on cell invasiveness in the five pancreatic cancer cell lines. SCF enhanced the invasive ability in the two KIT-positive cell lines (Fig. 3A). On the other hand, the invasive potential was not enhanced by SCF in the three KIT-negative cancer cell lines (Fig. 3B).

### Inhibitory effect of imatinib mesylate on proliferation and invasion

**Figure 4:**
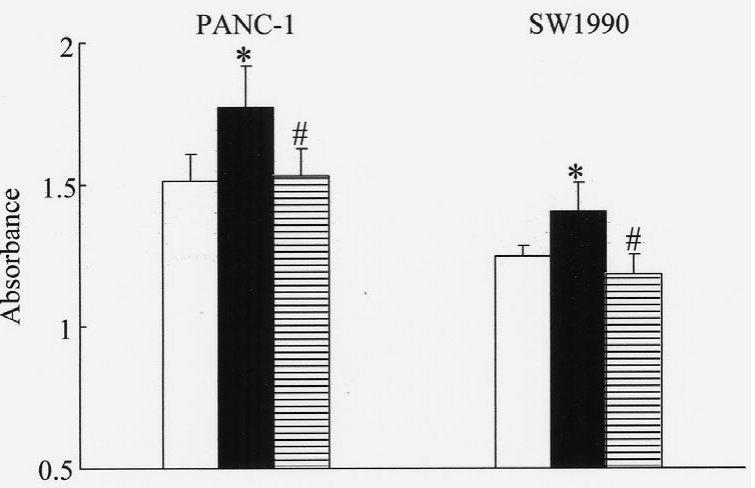
**Inhibitory effect of imatinib mesylate on SCF-enhanced proliferation in pancreatic cancer cell lines**. Cells were incubated without either SCF or imatinib mesylate (control, white columns), or with 10 ng/mL SCF (black columns), or with 10 ng/mL SCF and 5 μM imatinib mesylate (horizontal striped columns) for 72 hours and proliferation was measured by WST-1 assay. Statistical analysis was performed by one-way ANOVA with the SNK test. Bars indicate the SD, *, p < 0.05 compared with control; #, p < 0.01 compared with SCF 10 ng/mL.

**Figure 5:**
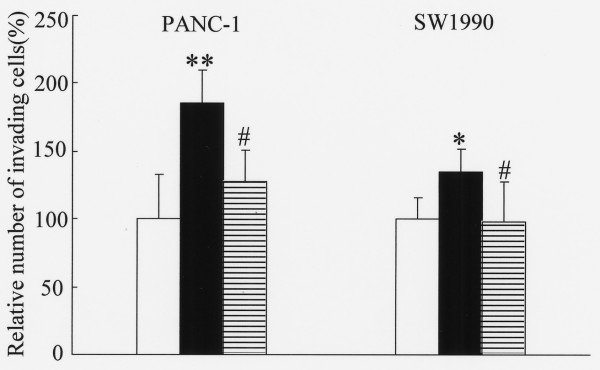
**Inhibitory effect of imatinib mesylate on SCF-induced invasive ability**. Cells were incubated without either SCF or imatinib mesylate (control, white columns), or with 10 ng/mL SCF (black columns) or with 10 ng/mL SCF and 5 μM imatinib mesylate (horizontal striped columns) for 24 hours. Statistical analysis was performed by one-way ANOVA with the SNK test. Bars indicate the SD *, p < 0.05 compared with control; **, p < 0.01 compared with control; #, p < 0.01 compared with SCF.

We asked whether imatinib mesylate might inhibit KIT-stimulated proliferative and invasive activities of the two KIT-positive pancreatic cancer cell lines (PANC-1 and SW1990). As shown in Fig. 4, 5 μM imatinib mesylate significantly inhibited SCF-enhanced proliferation to the level shown by nonstimulated control. Similarly, SCF-enhanced invasive ability was significantly inhibited by 5 μM imatinib mesylate treatment of PANC-1 and SW1990 (Fig. 5).

### Immunohistochemistry

**Figure 6:**
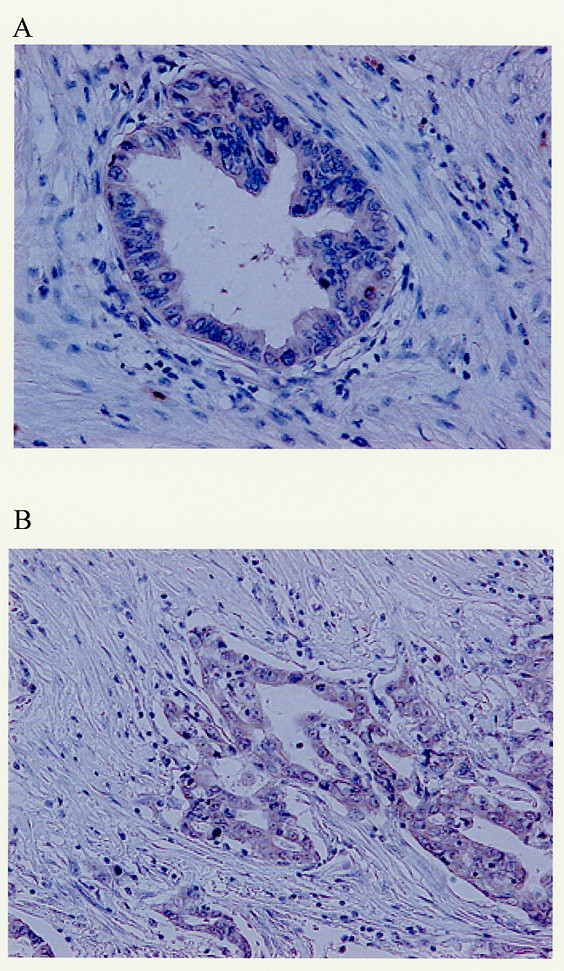
**Immunohistochemical detection of KIT and SCF expression in pancreatic cancer**. (A) KIT-positive pancreatic cancer cells (× 200). (B) SCF-positive pancreatic cancer cells (× 200).

**Figure 7:**
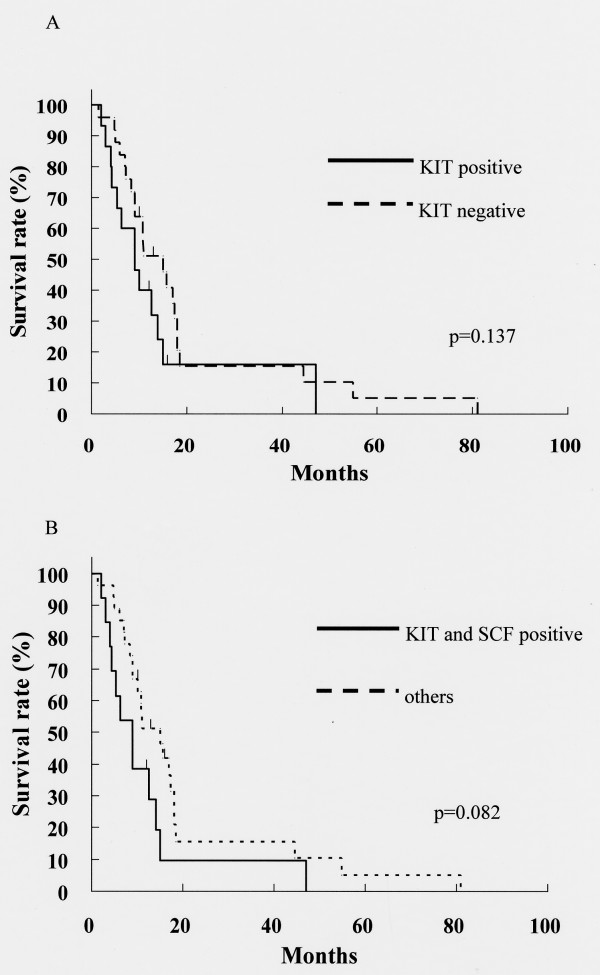
**Cumulative survival and expression of KIT and SCF**. Cumulative survival curve in (A) KIT-positive patients and (B) KIT-positive and SCF-positive patients by Kaplan-Meier analysis. Statistical significance was analyzed by the log-rank test.

KIT and SCF expression were evaluated in 42 patients with confirmed invasive ductal carcinoma of the pancreas. Fig. 6A shows one example of a KIT-positive pancreatic cancer cells and Fig. 6B shows one example of a SCF-positive pancreatic cancer cells. Table 1 shows the clinical and pathological characteristics of the patients. Pancreatic cancer cells expressed KIT in 16 of the 42 cases (38.1%). Characteristics of the patients, such as gender, age, location of the tumor, TNM stage and cancer cell differentiation, were not significantly different between the KIT-positive and KIT-negative groups. In lymph node metastases, lymphatic system invasion and intrapancreatic neural invasion, there were no significant differences between the two groups. But, the expression of KIT was correlated with venous system invasion (p = 0.046). Pancreatic cancer cells expressed SCF in fourteen KIT-positive specimens. In fact, fourteen specimens co-expressed KIT and SCF. Furthermore, KIT-positive specimens expressed SCF more frequently compared to KIT-negative specimens (p = 0.002). Concerning patient survival, KIT expression was not significantly correlated (Fig. 7A), but the cases co-expressing KIT and SCF had a tendency toward lower survival than others (Fig 7B). However, the difference was not statistically significant. The median follow-up time was 14.8 months after surgery.

**Table 1:** Clinical and pathological characteristics of patients according to the expression of KIT.

		KIT expression (overall n = 42)		
		
		KIT positive (n = 16)	KIT negative (n = 26)	p-value
Gender	Male/Female	10/6	18/8	NS
Age (year)		64.5 ± 7.2	66.3 ± 10.8	NS
TNM stage	I/II/III/IV/unclear	2/3/8/3/0	5/4/12/4/1	NS
Tumor location	Head/Body, Tail	10/6	22/4	NS
Cancer cell differentiation	W/Mo/P/Mu	3/11/1/1	11/10/5/0	NS
SCF	Positive/Negative	14/2	10/16	0.002
Lymph node metastasis	Positive/Negative	14/2	21/5	NS
Intrapancreatic neural invasion	ne2, 3/ne0, 1	14/2	17/9	NS
Lymphatic system invasion	ly2, 3/ly0, 1	9/7	13/13	NS
Venous system invasion	v2, 3/v0, 1	10/6	8/18	0.046

NS = not significant. W = well differentiated adenocarcinoma; Mo = moderately differentiated adenocarcinoma; P = papillary adenocarcinoma; Mu = mucinous carcinoma. Values of age are given mean ± SD. The p-values were obtained using the Mann-Whitney *U*-test.

## Discussion

In this study, we examined the relationship between c-*kit *expression and the malignant potential of pancreatic cancer cells. Thus, we demonstrated the influence of c-*kit *expression on proliferation and invasion of pancreatic cancer cell lines *in vitro*. In addition, we analyzed the relationship between KIT and SCF expression in pancreatic cancer tissues and examined clinical features and prognosis.

First, c-*kit *expression was examined in five pancreatic cancer cell lines. It was ascertained that two of the cell lines, PANC-1 and SW1990, expressed c-*kit *mRNA and protein by RT-PCR and Western blot analysis. Based on those results, we defined these two pancreatic cancer cell lines as KIT-positive. Next, we stimulated those lines with SCF to investigate the differences in proliferative responses between KIT-positive and KIT-negative cell lines. In the two KIT-positive cell lines, proliferation was significantly enhanced by SCF at concentrations above 1 ng/mL. On the other hand, SCF did not enhance proliferation of KIT-negative cancer cell lines. Results from the invasion (Matrigel) assay were consistent with the proliferation assay. Namely, the invasive ability of KIT-positive pancreatic cancer cell lines was significantly enhanced by addition of SCF at concentrations above 1 ng/mL. The fact that KIT activation induces proliferation or invasion *in vitro *was also reported in lung cancer and colorectal cancer [15,32,33]. In this study, 1 ng/mL SCF significantly enhanced proliferation and invasion. Because the human *in vivo *level of SCF in serum is between 1 ng/mL and 3 ng/mL [34-36], serum SCF concentration may be high enough to stimulate the proliferation and invasion of pancreatic cancer cells *in vivo*. Recently, it was shown that the existence of mast cells which showed immunoreactivity for SCF was ascertained in pancreatic cancer [31]. Our studies indicate that KIT expression by pancreatic cancer cells may have functional significance. Thus, we propose that serum SCF and SCF secreted from mast cells could contribute to *in vivo *tumor progression of KIT-positive pancreatic cancer.

In pancreatic cancer specimens, c-*kit *receptor was reportedly expressed in 6.1% based on immunohistochemical techniques [28]. On the other hand, two other studies from the same department reported that about 80% of pancreatic cancers expressed KIT [29,30]. In the present study, KIT was expressed in 38% of pancreatic cancer specimens (16 of 42 patients) by immunohistochemistry. In the report which concluded that 6.1% of pancreatic cancer expressed KIT, antigen retrieval was not performed. Thus, the percentage expressing KIT may be erroneously low. As to the studies reporting about 80% KIT expression, antigen retrieval was done using urea. On the other hand, we used Target Retrieval Solution High pH (DAKO, Copenhagen, Denmark) for antigen retrieval according to the antibody manufacturer's instruction. The difference between the methods relating to antigen retrieval may contribute to the differences in KIT-positive ratio.

In this study, clinicopathological findings associated with venous system invasion were significantly more severe in the KIT-positive group than in the KIT-negative group. Interestingly, KIT activation has been linked to vascular endothelial growth factor (VEGF) expression in GIST [37]. In addition, it was reported that VEGF expression was significantly correlated with venous invasion in gastric cancer [38]. From these facts, we suppose that KIT induced VEGF expression may have relationship with venous system invasion in pancreatic cancer. As to prognosis, in small-cell lung cancer, patients with KIT expression had a significant tendency toward lower survival than did KIT-negative patients [14]. Concerning pancreatic cancer, two studies reported that KIT-positive patients had a tendency toward lower survival than did c-kit-negative patients, but the differences were not significant [29,30]. Those results were confirmed here, as the difference in prognosis between KIT-positive and KIT-negative patients was not significant.

With regard to expression of SCF, pancreatic cancer specimens were evaluated for simultaneous expression with KIT. Co-expression of KIT and SCF has been reported in small-cell lung cancer, and in those diseases, autocrine pathways are suggested [39,40]. In our immunohistochemical study, 14 pancreatic cancer specimens co-expressed KIT and SCF, and the proportion of SCF-positive specimens was significantly larger in KIT-positive specimens than in KIT-negative ones. Patients co-expressing KIT and SCF had a tendency toward lower survival than others. Thus, autocrine pathways may play important roles in the evolution of malignant disease in the pancreas.

Recently, drugs which selectively inhibit tyrosine kinase receptors have been developed [24-26]. The goal is tumor-specific disruption of cell proliferation and differentiation. Targeted receptors include epidermal growth factor receptor and vascular endothelial growth factor receptor [41,42]. Because c-*kit *tyrosine kinase receptor has an important role in tumor progression, the KIT inhibitor imatinib mesylate is being explored for therapeutic efficacy [27]. Imatinib mesylate was initially designed to inhibit Bcr/Abl kinase which is present in 95% of patients with CML [43]. Later, it was found that imatinib mesylate also inhibited the c-*kit *tyrosine kinase receptor [44]. Imatinib mesylate is used for the therapy of GIST and leukemia and contributes to improvement of the prognosis, response ratio and quality of life [45]. In this study, we assessed the inhibitory effect of imatinib mesylate on proliferation and invasion of pancreatic cancer cells. In leukemia patients, imatinib mesylate plasma concentrations were in the range of 0.17 μM – 5.68 μM when 25 – 600 mg of the drug was administered per day [46]. We administered 5 μM imatinib mesylate to pancreatic cancer cell lines, and the concentration was within the *in vivo *range of plasma. In KIT-positive pancreatic cancer cell lines, 5 μM imatinib mesylate significantly inhibited SCF-enhanced proliferation to the level shown by nonstimulated control. Imatinib mesylate similarly reduced invasive potential. As to the therapy of pancreatic cancer, gemcitabine is used as the standard chemotherapy now, and therapies combining gemcitabine with other drugs have been explored [47]. For KIT-positive pancreatic cancers, KIT inhibitors could block the ligand-dependent signaling pathway. Thus, we propose that a KIT inhibitor could be used with gemcitabine, or used as a second choice when the pancreatic cancer is resistant to gemcitabine.

## Conclusion

KIT was expressed in 38% of pancreatic cancer patients and expression correlated with the degree of venous system invasion. Furthermore, KIT-positive specimens expressed significantly more SCF than did KIT-negative patients and patients expressing both KIT and SCF had a tendency toward lower survival. *In vitro*, we demonstrated that the SCF-KIT pathway enhanced the proliferation and invasiveness in KIT-positive pancreatic cancer cell lines and that the enhanced proliferation and invasion were inhibited by imatinib mesylate. We propose that inhibitors of c-*kit *tyrosine kinase receptor have the potential to slow the progression of KIT-positive pancreatic cancers. Thus, clinical exploration of such inhibitors in the setting of pancreatic cancer should be pursued.

## Methods

### Cell culture

Five human pancreatic cancer cell lines (SW1990, PANC-1, MIA PaCa-2, Capan2 and BxPC3) were obtained from the American Type Culture Collection (Rockville, MD). SW1990, PANC-1 and MIA PaCa-2 were maintained in Dulbecco's modified Eagle's medium (Sigma Chemical Co., St. Louis, MO) supplemented with 10% fetal bovine serum. Capan2 was maintained in McCoy's medium supplemented with 10% fetal bovine serum. BxPC3 was maintained in RPMI-1640 medium (Sigma Chemical Co., St. Louis, Mo) supplemented with 10% fetal bovine serum. All cells were incubated at 37°C in a humidified atmosphere of 5% CO_2 _in air.

### Reagents and antibodies

Recombinant human SCF was purchased from R&D Systems (Abingdon, UK). For Western blot analysis, rabbit polyclonal anti-KIT antibody was obtained from Med. & Biological Laboratories (Nagoya, Japan) and rabbit monoclonal anti-β-actin antibody was obtained from Cell Signaling Technology (Beverly, MA). For immunohistochemistry, rabbit polyclonal anti-KIT antibody and rabbit polyclonal anti-SCF antibody were purchased from Immuno-Biological Laboratories Co., Ltd. (Gunma, Japan). KIT inhibitor imatinib mesylate was kindly provided by Novartis Pharma AG (Basel, Switzerland).

### RT-PCR analysis

Total RNA was extracted from five pancreatic cancer cell lines using Isogen Kits (Nippon Gene Tokyo, Japan), and quantities determined spectrophotometrically. Total RNA aliquots (1 μg) were pretreated with Dnase I (Boehringer Mannheim, Germany) for 20 minutes at room temperature, denatured at 70°C for 10 minutes, chilled on ice, and then reverse-transcribed into cDNA in a reaction mixture containing 10 mmol/L dithiothreitol, 0.5 mmol/L dNTPs, first standard buffer, and 1U Superscript II (Invitrogen, San Diego, CA) at 42°C for 60 minutes. Reactions were terminated by heating at 72°C for 10 minutes. Reaction mixture aliquots (1 μL) were used as templates for PCR. The following primers for c-*kit *were used: forward 5'-gcccacaatagattggtattt-3' and reverse 5'-agcatctttacagcgacagtc-3'. The PCR product size was 570 bp. The PCR conditions were as follows: denaturation at 94°C for 4 minutes, annealing at 51°C for 1 minute, extension at 72°C for 1 minute, followed by 34 cycles of denaturation at 94°C for 1 minute, annealing at 51°C for 1 minute, and extension at 72°C for 1 minute. The final extension was at 72°C for 7 minutes. The PCR products were separated and detected using ethidium-bromide stained 1.5% agarose gels.

### Western blot analysis

Total cell lysates from confluent cultures were prepared using ice-cold radioimmunoprecipitation assay (RIPA) buffer containing leupeptin, aprotinin, and pepstein A. Protein concentration of the cell lysates was measured using a BCA protein assay kit (Pierce, Rockford, IL). 30 μg of protein from cell lysates were separated on 7.5% SDS-PAGE and transferred to a PVDF membrane. The membrane was incubated in blocking buffer for 2 hours at room temperature. The blocking buffer consisted of 5% nonfat dry milk in Tris buffered saline containing 0.1% Tween 20 (TBS-T). After washing the membrane with TBS-T, the membrane was incubated with rabbit anti-KIT antibody diluted 1:1000 overnight at 4°C. Then, the membrane was washed with TBS-T, and subjected to horseradish peroxidase-conjugated anti-rabbit immunoglobulin (DAKO, Copenhagen, Denmark) diluted 1:2000 as a second antibody for 1 hour at room temperature. Protein-antibody complexes were visualized with an ECL Western blotting detection and analysis system (Amersham Biosciences, Buckinghamshire, UK). The membrane was washed and stripped using Restore Western Blot Stripping Buffer (Pierce, Rockford, IL) and β-actin was detected using rabbit anti-β-actin antibody by the same method.

### Proliferation assay

To examine the influence of SCF on cell proliferation, we used the Premix WST-1 Cell Proliferation Assay System (TAKARA BIO INC., Shiga, Japan). Five human pancreatic cancer cell lines were seeded at a density of 3 × 10^3^/100 μL in 96-well plates and allowed to adhere for 24 hours. Then, cultures were refed with fresh media containing various concentration of SCF. After 72 hours incubation, 10 μL WST-1 reagent was added to each well and the trays were incubated for 2 hours at 37°C, at which point the absorbance was measured using a microplate reader with a test wave length of 450 nm and a reference wavelength of 690 nm. In studies of imatinib mesylate, cells were incubated with SCF (10 ng/mL) alone or with both SCF (10 ng/mL) and imatinib mesylate (5 μM). In controls, cells were incubated without either SCF or imatinib mesylate. After 72 hours incubation, the inhibitory effect was evaluated using the WST-1 Cell Proliferation Assay System

### Invasion assay

The *in vitro *invasive potential of human pancreatic cancer cells was determined using BioCoat Matrigel Invasion Chambers (Becton Dickinson, Bedford, MA). This system is separated by a PET membrane coated with Matrigel Matrix such that only invasive cells can migrate through the membrane to the reverse side. After rehydration for 2 hours in a humidified incubator at 37°C with 5% CO_2_, cells were suspended in medium containing 10% FBS, placed in the upper chamber at a concentration of 1 × 10^5 ^cells/chamber and incubated for 24 hours at 37°C with 5% CO_2 _with various concentrations of SCF. After 24 hours incubation, noninvasive cells were removed from the upper surface of the membrane by scrubbing. The cells on the reverse side of the membrane were fixed with 70% ethanol, stained with Giemsa solution, and counted under a microscope at 200 × magnification. We also investigated the inhibitory effect of imatinib mesylate on SCF-mediated invasion using the same method. Namely, cells were incubated with SCF (10 ng/mL) or with both SCF (10 ng/mL) and imatinib mesylate (5 μM) for 24 hours. For controls, cells were incubated without either SCF or imatinib mesylate.

### Immunohistochemistry

Forty-two patients diagnosed with invasive ductal adenocarcinoma of the pancreas underwent pancreatic resections at Nagoya City University Hospital between April 1989 and March 2005 and were included in this study.

Specimens were fixed in 10% formalin and then embedded in paraffin. Specimens were sectioned into 3 μm-thick slices and the sections were deparaffinized. Next, the sections were subjected to autoclave treatment in Target Retrieval Solution High pH (DAKO, Copenhagen, Denmark) for 20 minutes at 95°C for KIT antigen retrieval. For SCF, the sections were subjected to autoclave treatment in 0.01 M sodium citrate buffer (pH 6.0) for 20 minutes at 95°C. Then, all sections were immersed in blocking reagent (Block Ace, Dainippon Pharma Co., Ltd., Osaka, Japan) for 10 minutes to reduce non-specific binding. After removal of excess serum, the sections were incubated with primary antibodies against KIT or SCF (diluted 1:50) for 60 minutes at room temperature. After rinsing in phosphate buffered saline (PBS), the sections were treated with horseradish peroxidase-labeled anti-rabbit immunoglobulin (DAKO, Copenhagen, Denmark) as a second antibody for 30 minutes at room temperature. The peroxidase reaction was visualized by incubating the sections with 0.02% 3.3'-diaminobenzidine tetrahydrochloride in 0.05 M Tris buffer (pH 7.6) with 0.01% hydrogen peroxide, followed by hematoxylin counter staining. As a negative control, staining was also performed using normal mouse immunoglobulin G in place of the primary antibody. Results of each immunohistochemical staining were evaluated as follows. The intensity of tissue staining was graded semiquantitatively on a four-point scale (-, +, ++, +++), and the proportion of cells stained was assessed on a four-tier scale (1. 0%-15%; 2. 15%-50%; 3. 50%-85%; and 4. 85%-100% cells stained). The cases were classified into positive group and negative group by the intensity and proportion of immunostained cancer cells for KIT and SCF. Cases where the immunostaining intensity was more than ++ and the proportion was more than 2 were defined as positive group. When venous system invasion was evaluated, we classified v0 and v1 as the slight group, and v2 and v3 as the severe group. We assessed the relationship between KIT expression and two groups of venous invasion. With regard to intrapancreatic neural invasion and lymphatic system invasion, evaluations were conducted in the same way as venous invasion.

### Statistical analysis

All experiments were performed in triplicate. Results are expressed as means ± SD (standard deviation). Multiple group comparisons were performed by one-way ANOVA with a post hoc test for subsequent individual group comparison. Differences between two samples were analyzed by an unpaired *t *test. Survival analyses were calculated by using the Kaplan-Meier method and the differences in survival among subjects were compared with the log-rank test. The Mann-Whitney *U*-test was used to compare the immunohistochemical characteristics. Differences were considered statistically significant at p < 0.05.

## Abbreviations

GIST, gastrointestinal stromal tumor; CML, chronic myelogenous leukaemia; SCF, stem cell factor; VEGF vascular endothelial growth factor; SDS-PAGE, SDS-polyacrylamide gel electrophoresis; TBS, tris buffered saline; PBS, phosphate buffered saline.

## Competing interests

The author(s) declare that they have no competing interests.

## Authors' contributions

AY carried out the proliferation assay and immunohistochemical study in addition to the drafting of the manuscript. HS participated in the Western blots and immunohistochemical study. HT and YM performed the cell culture and the Western blots. NO and HF participated in the invasion assay and statistical analyses. MS and YO contributed RT-PCR and the literature search. HT designed the experiments and contributed to the writing of the manuscript. TM conceived the project and aided in experimental design. All authors read and approved the final manuscript.
